# Airborne imaging spectroscopy surveys of Arctic and boreal Alaska and northwestern Canada 2017–2023

**DOI:** 10.1038/s41597-025-04898-w

**Published:** 2025-04-25

**Authors:** Charles E. Miller, Robert O. Green, David R. Thompson, Andrew J. Thorpe, Michael L. Eastwood, Ian B. McCubbin, Winston Oslon-Duvall, Michael A. Bernas, Charles M. Sarture, Luis M. Rios, M. A. Hernandez, Brian D. Bue, Sarah R. Lundeen, Ryan Pavlick, John W. Chapman, Philip G. Brodrick, Regina F. Eckert, R. Willow Coleman, Latha Baskaran, Clayton D. Elder, Philip A. Townsend, Kyle R. Kovach, Shawn P. Serbin, Karl F. Huemmrich, Peter R. Nelson, Uma Bhatt, Matthew J. Macander, Debjani Singh, Michele Thornton, Daryl Yang, Isla Myers-Smith, Scott J. Goetz, Elizabeth E. Hoy, Elizabeth Larson, Dan Hodkinson, Hank A. Margolis, Michael Falkowski, Andrew Applejohn, Peter C. Griffith

**Affiliations:** 1 Jet Propulsion Laboratory, California Institute of Technology, Pasadena, California USA; 2 NASA Headquarters, Washington, DC USA; 3 NASA Ames Research Center, Moffet Field, California, USA; 4 Earth Science Division, NASA Ames Research Center, Moffett Field, USA; 5 University of Wisconsin, Madison, Wisconsin USA; 6 NASA Goddard Space Flight Center, Greenbelt, Maryland USA; 7 Schoodic Institute at Acadia National Park, Winter Harbor, Maine, USA; 8 Department of Atmospheric Sciences, University of Alaska Fairbanks, Fairbanks, Alaska USA; 9 ABR, Inc, Fairbanks, Alaska USA; 10 Biological and Environmental Systems Science Directorate, Oak Ridge National Laboratory, Oak Ridge, Tennessee USA; 11 University of British Columbia, Vancouver, British Columbia Canada; 12 School of Informatics, Computing, and Cyber Systems, Northern Arizona University, Flagstaff, Arizona USA; 13 NASA Goddard Space Flight Center / GST, Inc., Greenbelt, Maryland USA; 14 NASA Goddard Space Flight Center / SSAI, Greenbelt, Maryland USA; 15 Government of the Northwest Territories, Yellowknife, Northwest Territories Canada; 16 Polar Knowledge Canada/Savoire Polaire, Ottawa, Ontario Canada

**Keywords:** Ecosystem ecology, Biodiversity, Forest ecology

## Abstract

Since 2015, NASA’s Arctic Boreal Vulnerability Experiment (ABoVE) has investigated how climate change impacts the vulnerability and/or resilience of the permafrost-affected ecosystems of Alaska and northwestern Canada. ABoVE conducted extensive surveys with the Next Generation Airborne Visible/Infrared Imaging Spectrometer (AVIRIS-NG) during 2017, 2018, 2019, and 2022 and with AVIRIS-3 in 2023 to characterize tundra, taiga, peatlands, and wetlands in unprecedented detail. The ABoVE AVIRIS dataset comprises ~1700 individual flight lines covering ~120,000 km^2^ with nominal 5 m × 5 m spatial resolution. Data include individual transects to capture important gradients like the tundra-taiga ecotone and maps of up to 10,000 km^2^ for key study areas like the Mackenzie Delta. The ABoVE AVIRIS surveys enable diverse ecosystem science, provide crucial benchmark data for validating retrievals from the PACE, PRISMA, and EnMAP satellite sensors and help prepare for the SBG and CHIME missions. This paper guides interested researchers to fully explore the ABoVE AVIRIS spectral imagery and complements our guide to the ABoVE airborne synthetic aperture radar surveys.

## Background & Summary

**Fig. 1:**
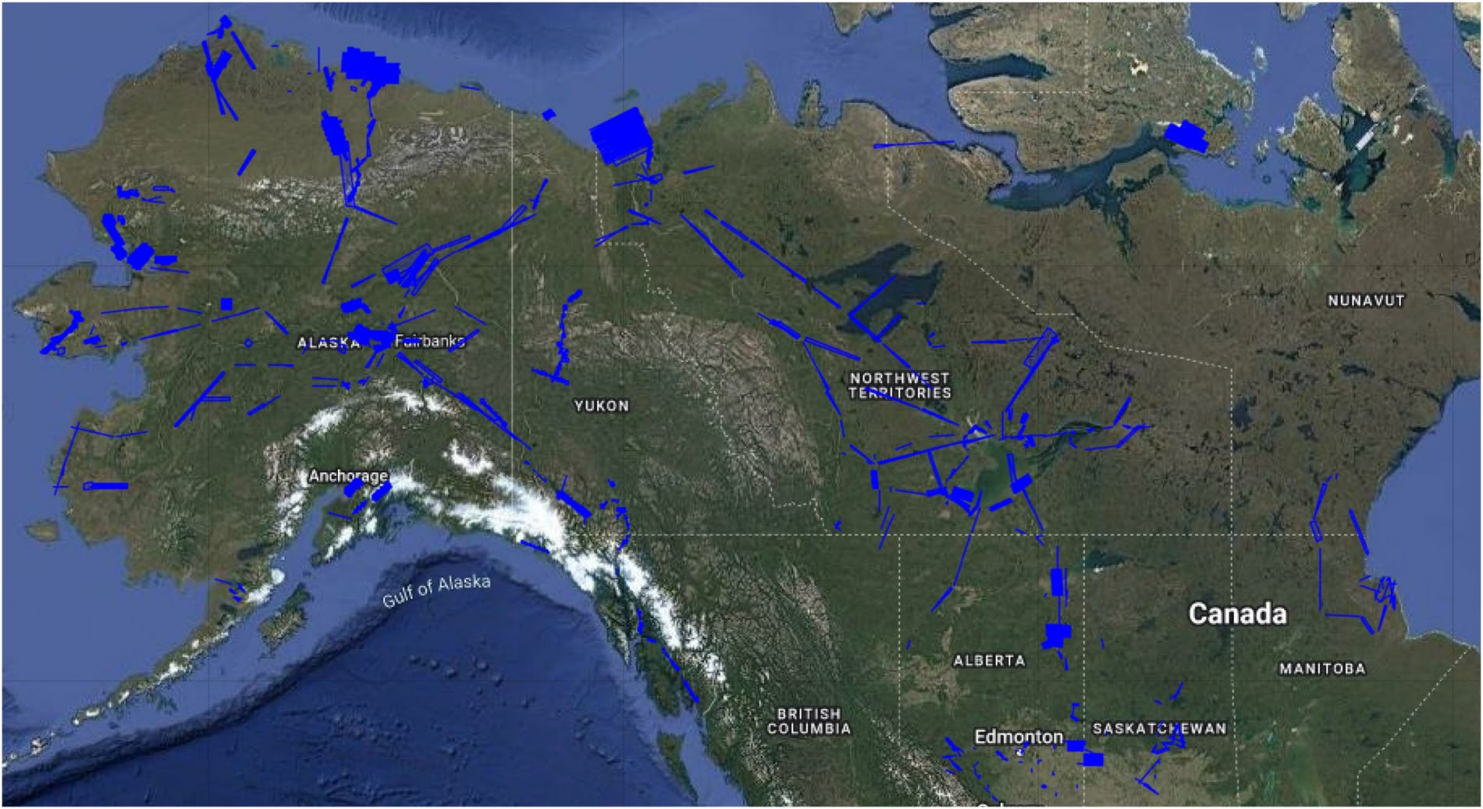
The ABoVE AVIRIS airborne surveys include more than 1700 individual flight lines collected from 2017 to 2023 across Alaska and northwestern Canada. Image created using the AVIRIS-NG data portal (https://avirisng.jpl.nasa.gov/dataportal/).

Arctic amplification, the warming of the Arctic nearly four times faster than the global mean^1,2^, has accelerated change in the northern high latitudes, leading to the “borealization” of the tundra^3^ and rapid transformation of permafrost-affected ecosystems^4–6^. Since 2017, NASA’s Arctic-Boreal Vulnerability Experiment (ABoVE) Science Team has executed airborne surveys over ~4 million km^2^ of Alaska and northwestern Canada with the objective of creating an interannual time series to determine whether Arctic ecosystems are vulnerable or resilient to this change^7^. ABoVE conducted extensive airborne surveys with the Next Generation Airborne Visible/Infrared Imaging Spectrometer (AVIRIS-NG) during 2017, 2018, 2019, and 2022 and with AVIRIS-3 in 2023 to characterize tundra, taiga, peatlands, and wetlands in unprecedented detail (Fig. 1). The surveys targeted vegetated surfaces and wetlands characteristic of the diverse ecoregions within the ABoVE domain. This benchmark high latitude data record is crucial to developing global imaging spectroscopy retrieval algorithms for NASA’s Surface Biology and Geology (SBG) mission^8,9^ and ESA’s Copernicus Hyperspectral Imaging Mission for the Environment (CHIME) mission^10^ due to (1) High signal-to-noise ratios, (2) Nominal ~5 m × 5 m spatial resolution, (3) Extended 7-year duration, and (4) Limitations of the International Space Station orbit dynamics preclude any data acquisitions north of 52 N by NASA’s Earth Surface Mineral Dust Source Investigation (EMIT), a precursor of the AVIRIS-3 and SBG instruments^11,12^. Additionally, the ABoVE AVIRIS surveys will help validate challenging high-latitude terrestrial and coastal retrievals from NASA’s Plankton, Aerosol, Cloud, ocean Ecosystem (PACE) mission^13^, the Italian Space Agency’s Hyperspectral Precursor of the Application Mission (PRISMA)^14^ and the German Space Agency’s Environmental Mapping and Analysis Program (EnMAP)^15^.

The ABoVE AVIRIS imaging spectroscopy data spatially overlap with and complement other ABoVE Airborne data(Table 1) including: L-band and P-band polarized interferometric synthetic aperture radar (PolInSAR)^1^; LVIS full-waveform lidar^16^; AirSWOT Ka-band water surface elevation surveys^17^; and the Arctic-CAP time series of *in situ* CO_2_ and CH_4_ vertical profile surveys^18^. The planned collocation of data from diverse airborne sensors enables multi-sensor syntheses as precursors for similar studies using current and planned satellite sensors. For example, water surface elevations derived jointly from AirSWOT and LVIS^19^ or vegetation type and cover retrieved from AVIRIS imaging spectroscopy can be combined with surface classification and inundation state information from L-band PolInSAR to map large wetlands complexes^20^.

**Table 1:** Ancillary Airborne Sensors Deployed During the ABoVE Airborne Campaigns^7^.

Sensor	Description	Link
UAVSAR	L-band and P-band polarized interferometric synthetic aperture radar (PolInSAR)	https://uavsar.jpl.nasa.gov/cgi-bin/data.pl
AirSWOT	Ka-band water surface elevation	https://swot.jpl.nasa.gov/mission/airswot/
LVIS	Full waveform lidar	https://lvis.gsfc.nasa.gov/Data/Maps/ABoVE2019Map.html
Arctic-CAP	*In situ* CO_2_, CH_4_ and CO vertical profiles	10.5194/acp-22-6347-202

**Fig. 2:**
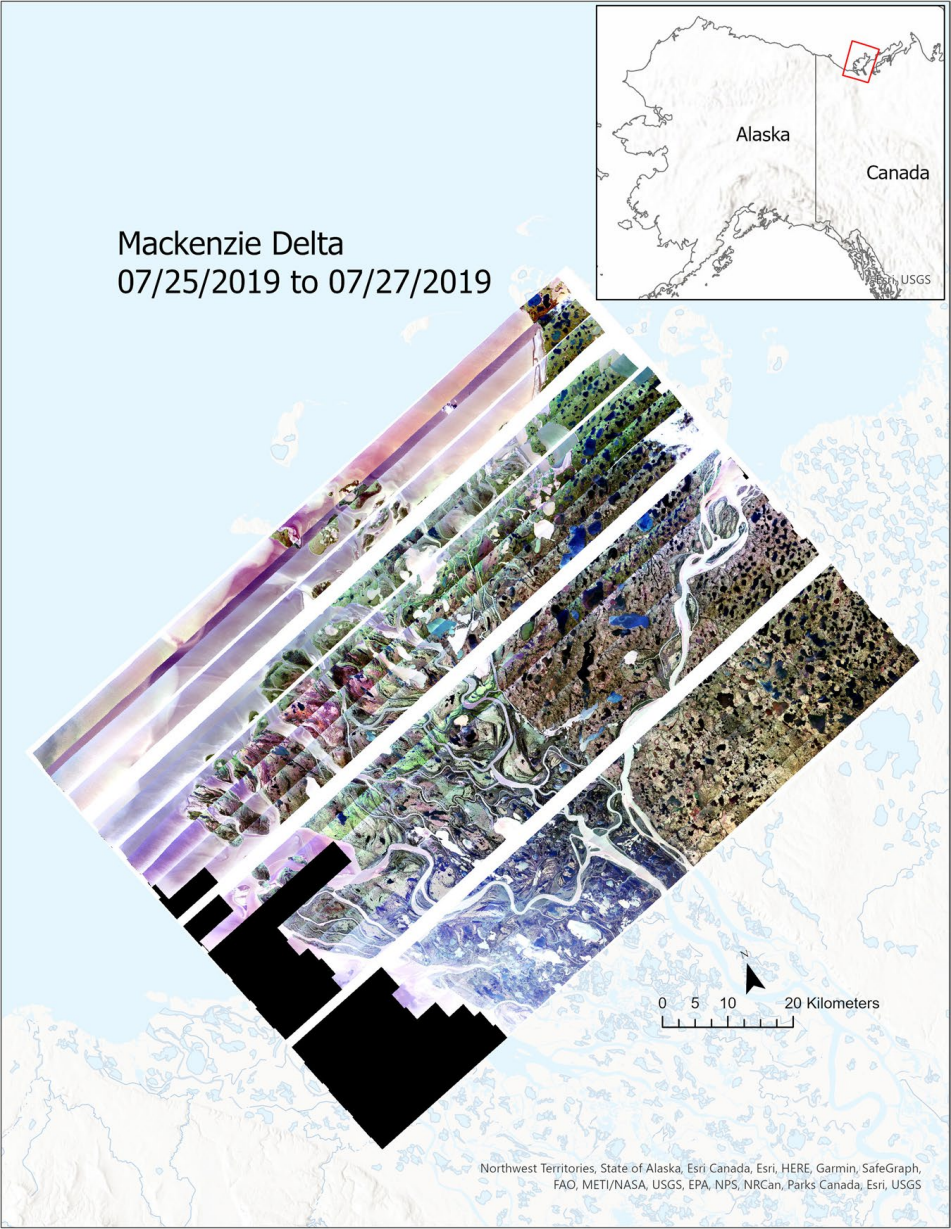
AVIRIS-NG map of the Mackenzie Delta captures the complex interplay of hydrology and vegetation throughout the Delta as well as the impact of sediment loading on the major channels and the Beaufort Sea coastal region. The map is a composite of more than 30 different 100-km long transects acquired from 25–27 July 2019. Individual pixels have a spatial resolution of ~25 m^2^ and the total area of the map is ~10,000 km^2^, enabling researchers to investigate scaling phenomena and emergent properties over eight orders of magnitude^28^.

**Fig. 3:**
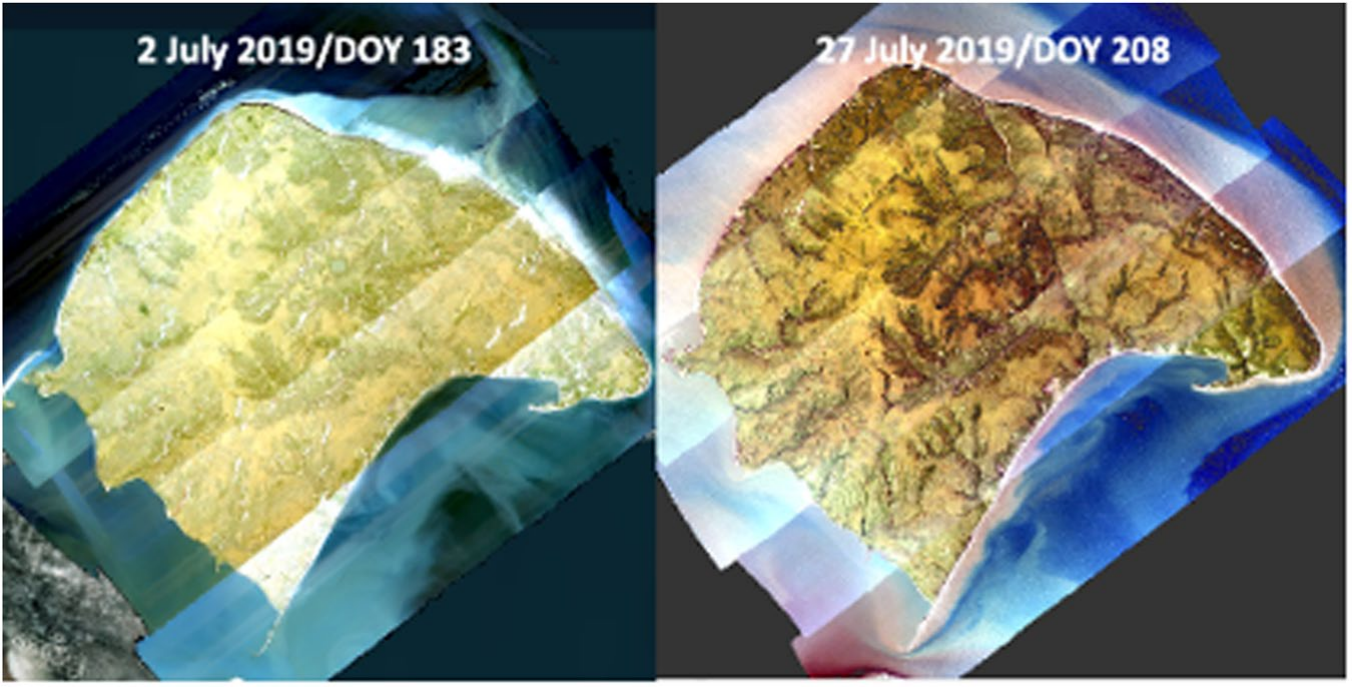
AVIRIS-NG maps of Qikiqtaruk, YT (Herschel Island) reveal the rapid greening that occurred during July 2019 [Kerby and Myers-Smith, unpubl].

**Fig. 4:**
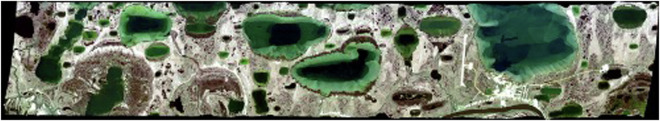
Detail from an AVIRIS-NG acquisition over Atqasuk, AK on 29 July 2018 illustrates the variety of small, large, merged, and drained thermokarst lakes carpeting the region. The 1200-m airstrip and Atqasuk village are visible on the right-hand side of the image. Vegetation species characterization and ground-based spectra have also been acquired for validation of the airborne retrievals^58^.

ABoVE AVIRIS data are delivered as calibrated, orthorectified, geolocated reflectances that have been corrected for atmospheric effects. The reflectance data enable researchers to generate higher level data products such as vegetation traits and composition maps, water quality, and fractional cover. Continuous maps (Figs. 2, 3) with areas of up to 10,000 km^2^ at 25 m^2^ sampling enable researchers to investigate spatial scaling processes over eight orders of magnitude. Many of the transects sampled over long-term monitoring stations or locations where field teams collected same day vegetation samples to ensure robust validation of the higher-level data products (Fig. 4). This includes sampling of foliar material and plant composition data from field plots coincident with the AVIRIS-NG spectral imagery acquisitions by teams interested in scaling up ground-based plant and community properties. Field teams measured biochemical and morphological plant properties both *in-situ* and in the laboratory for use in developing algorithms to retrieve plant foliar functional traits from AVIRIS-NG imagery. These trait estimations made on the pixel level of the airborne imagery encompass 28 plant foliar functional traits with associated uncertainties^21^.

The ABoVE AVIRIS-NG surveys already enabled a wide range of investigations. Examples include assessing boreal forest wildfire fuel loading^22–25^ as well as identifying methane emissions hotspots from wetlands^26–28^, beaver hydraulic engineering^29^, tundra fire burned areas^30^, and tar sands oil production facilities^31^. The ABoVE team has made significant progress comparing vegetation classifications and traits derived from AVIRIS-NG with the values retrieved from centimetre-resolution uncrewed aerial vehicle (UAV) sensors^32,33^. AVIRIS-NG observations were also collected over tower sites that supported automated spectrometers collecting time series of spectral reflectance and solar induced fluorescence (SIF) linking descriptions of diurnal and seasonal optical variability with the imagery^34,35^. Many additional studies and multi-sensor synthesis efforts are underway as part of ABoVE synthesis and analysis activities.

## Methods

The ABoVE AVIRIS surveys represent one of the largest airborne imaging spectroscopy data records collected to date, complementing the Western Diversity Time Series (California) [https://impact.earthdata.nasa.gov/casei/campaign/WDTS/], the AVIRIS-India campaigns^36,37^, and the California Methane Surveys^38^. Here, we describe the AVIRIS sensors, the strategy behind the ABoVE AVIRIS data acquisitions, and the resulting data records.

### AVIRIS-NG

The Airborne Visible/Infrared Imaging Spectrometer-Next Generation (AVIRIS-NG) has been described in detail previously^39^. Briefly, AVIRIS-NG measures reflected radiance in 425 bands at 5 nm intervals in the visible to shortwave infrared spectral range between 380 and 2510 nm. AVIRIS-NG contains 600 cross-track imaging pixels, each having a 1 milliradian field of view, resulting in ~5 m ground sampling and ~ 3000 m swath from the typical 5000 m above ground level (AGL) flight altitude. Each spatial pixel returns a fully resolved spectrum. The resulting data cubes are stored onboard for processing after each flight. Dark calibration scans are recorded at regular intervals during each flight. Measurements are radiometrically and geometrically calibrated. Table 2 summarizes the AVIRIS-NG instrument characteristics.

**Table 2:** Spectral, Radiometric, Spatial, and Uniformity Properties of AVIRIS-3 and AVIRIS-NG.

Spectrometer	AVIRIS-3	AVIRIS-NG
**Spectral**		
Range	380–2510 nm	380–2510 nm
Sampling	7.4 nm	5 nm
Response	1.0–1.5x sampling	1.0–1.5x sampling
Calibration	±0.01 nm	±0.01 nm
**Radiometric**		
Range	0 - max Lambertian	0 - max Lambertian
Precision (SNR	>3000 @ 600 nm	>2000 @ 600 nm
	>1200 @ 2200 nm	>1000 @ 2200 nm
Calibration	97% (<3% unc)	95% (<5% unc)
**Spatial**		
Swath Samples	1240	600
Swath Angle	40 deg FOV	34 deg FOV
Sampling	0.6 mrad	1 mrad
Repsonse (FWHM)	1–1.5x sampling	1–1.5x sampling
Ground Sample	3 m @ 5000 m alt	5 m @ 5000 m alt
**Uniformity**		
Spectral Cross-Track	>95% across FOV	>95% across FOV
Spectral-IFOV Variation	>95% Spectral direction	>95% Spectral direction

### AVIRIS-3

Airborne Visible/Infrared Imaging Spectrometer 3 (AVIRIS-3) is the third of the NASA AVIRIS spectrometer series^40^. The core spectrometer of AVIRIS-3 is a copy of the optically fast, F/1.8 Dyson imaging spectrometer used by EMIT and installed on the International Space Station (ISS) in 2022. AVIRIS-3 uses the EMIT spectrometer design interfaced with a scaled two-mirror telescope enclosed in a compact vacuum vessel to enable measurements from airborne platforms ranging from a Twin Otter to a Gulfstream business jet or a NASA ER-2. AVIRIS-3 is a cryogenic instrument with advanced system control and real-time onboard spectroscopic data processing algorithms evolved from AVIRIS-NG. The spectral range of AVIRIS-3 is 380 to 2500 nm with 7.4 nm sampling. The radiometric range is from 0 to max terrestrial Lambertian radiance with higher signal-to-noise ratio performance than AVIRIS-Classic or AVIRIS-Next Generation. The spatial field-of-view is 39.5 degrees with 0.56 milliradian sampling. Table 2 compares the instrument properties of AVIRIS-3 to those of AVIRIS-NG^40^.

### ABoVE AVIRIS Airborne Surveys

ABoVE is a NASA Terrestrial Ecology Program decade-long community field experiment designed to understand the vulnerability and resilience of ecosystems and society to rapid change in the Arctic-boreal region of Alaska and northwestern Canada. To date, the ABoVE Science Team has integrated more than 75 Principal Investigator led projects addressing multiple themes, including (with number of NASA-funded projects in parentheses): pre-ABoVE investigations (5), Ecosystem Dynamics (19), Ecosystem Services (21), Airborne Science (10), and Synthesis and Analysis (20). Additionally, there are Affiliated (34) and Partner (2) projects as well as projects sponsored by other NASA programs (50).

The ABoVE AVIRIS flight lines were designed as part of the integrated ABoVE airborne campaign strategy^7^. This strategy targeted acquisitions to support ABoVE Science Team investigations, long-term monitoring stations, recently disturbed areas, and priorities defined during consultations with local stakeholders across the domain. Additionally, numerous lines were specifically designed to overlap with acquisitions planned for the L-band and P-band synthetic aperture radar (SAR) instruments^1^, the airborne validation sensor for the Surface Water and Ocean Topography mission (AirSWOT)^17^, and full waveform lidar from the Land, Vegetation and Ice Sensor (LVIS)^16^. A key benefit of ABoVE Science Team membership was that all projects were empowered to request AVIRIS acquisitions over their targets. Requests ranged from individual ground sites to 10,000 km^2^ maps to transects across the entire ABoVE domain. These requests were gathered annually, entered into the AVIRIS data acquisition system, and prioritized. Flights each year balanced repeat acquisitions over key long-term ground sites or previously viewed scenes to capture ecosystem response/recovery time series and acquisitions over new sites, and pickups of acquisitions that were not possible in previous years due to weather or wildfire smoke. We typically acquired 300 individual lines (>25,000 km^2^) per year.

AVIRIS spectral imaging surveys of the ABoVE domain were conducted from June to August in 2017, 2018, 2019, 2022, and 2023 (Fig. 1). AVIRIS-NG or AVIRIS-3 was integrated on a King Air B-200 (Dynamic Aviation, N53W) onto a nadir viewing port. Flights were timed to coincide with maximum solar elevation to maximize signal-to-noise ratios, with takeoff typically between 0900 and 1000 local time. Local flights lasted ~4 hours while sorties to more distant targets lasted up to 8 hours and involved stopping to refuel.

The ABoVE surveys operated primarily out of Fairbanks, AK or Yellowknife, NT. Short-term deployments were also made from Inuvik, NT, Cambridge Bay, NU, Saskatoon, SK, and Churchill, MB for data collections in these areas. Daily sorties were selected based on target priorities, whether there were field teams deployed at a given location, and the simultaneous requirements for clear-skies (>90% cloud free) and no/minimal ground level wildfire smoke. Persistently cloudy conditions over targets like Utqiagvik, AK, the Yukon-Kuskokwim Delta, and the Mackenzie Delta prioritized acquisitions at these locations when there were high confidence forecasts for clear skies. Wildfire smoke during high-fire years proved exceptionally challenging, resulting in poor data yields in Alaska in 2022 and preventing any acquisitions in Canada east of Whitehorse, YT in 2023.

### AVIRIS Data Processing Workflow

**Fig. 5:**
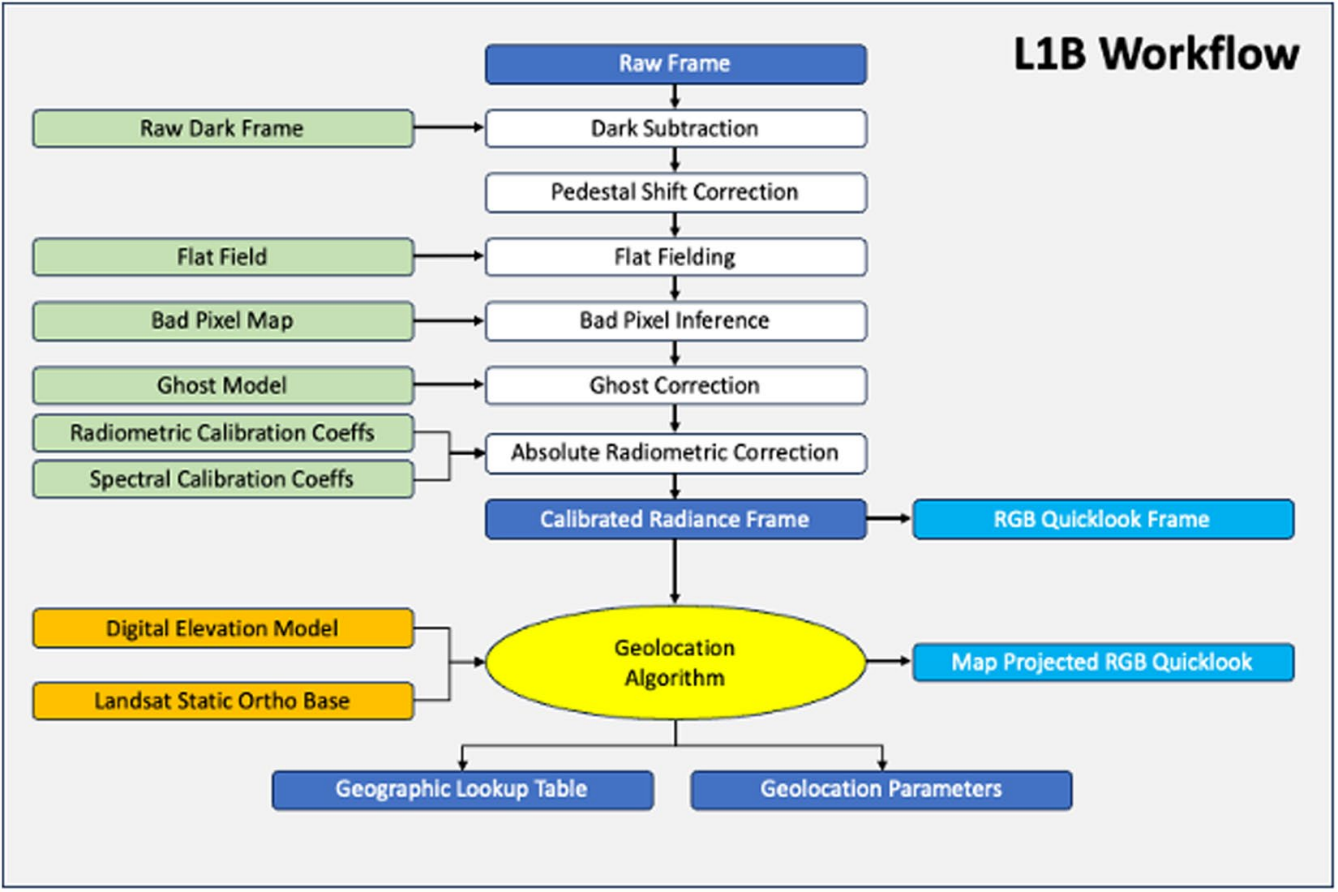
The AVIRIS-NG and AVIRIS-3 Level 1B data processing workflows leverage the recent advances made in processing EMIT data^11^ as well as decades of experience from flying AVIRIS sensors^41,42^.

**Fig. 6:**
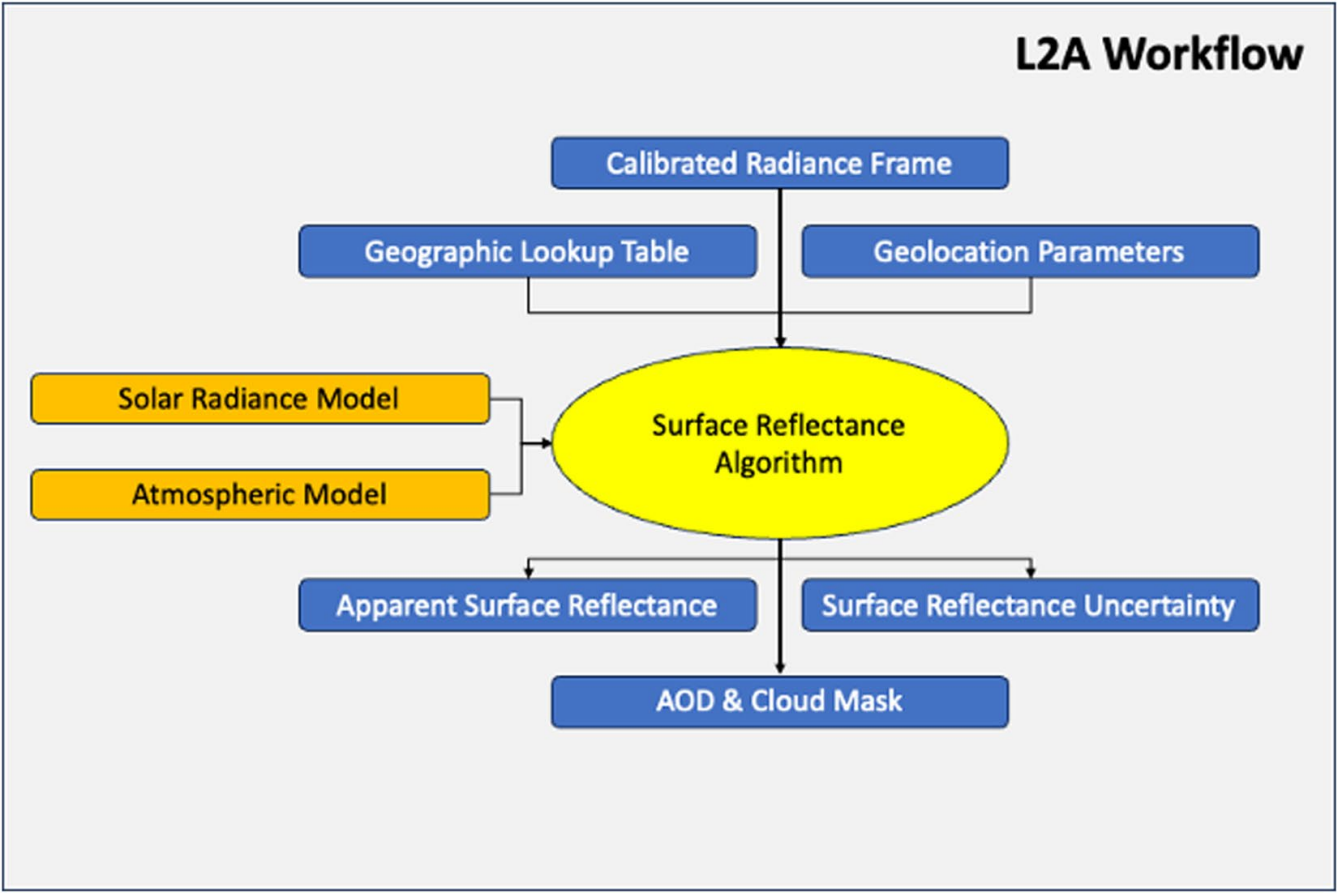
The AVIRIS-NG and AVIRIS-3 Level 2A data processing workflows leverage the recent advances made in processing EMIT data^12^ and optimizing the atmospheric correction scheme^43,46^.

The data processing workflows used to convert the raw image cubes into Level 1B calibrated radiances have been described in detail previously for both AVIRIS-NG^41^ and AVIRIS-3^42^. The ABoVE Level 2A reflectance products for all years (2017–2023) have been consistently processed based on the recent advances made by the AVIRIS team in processing EMIT spectral imagery^11,12^, particularly atmospheric correction using the open-source ISOFIT code^43^. Figure 5 summarizes the AVIRIS L1B data processing workflow while Fig. 6 presents the L2A data processing workflow.

### Level 1B Calibrated Radiances

Experience from many years of airborne surveys has demonstrated that AVIRIS calibration products derived from in-flight data result in the highest-fidelity surface reflectance spectra with the fewest atmospheric artifacts even though all inputs to the L1B processing pipeline can be derived from laboratory measurements^44^. Therefore, the dark frame, flatfield, bad pixel map, radiometric calibration coefficients, and spectral calibration are all updated using inflight data (Fig. 5).

We obtain the dark frame from the median of several thousand frames captured with a light-blocking shutter immediately before and after each flightline. We derive column- and row-pedestal shift corrections for each frame using masked rows and columns on the focal plane array. The pedestal shift corrects for electronic effects created by varied illumination levels across the focal plane array.

We multiply the dark-corrected frame by the flatfield to adjust for the slightly different responsivity of each pixel. We obtain the initial flatfield in the hangar by scanning a broadband light source across the entire field of view. We update the flatfield by capturing inflight data over bright, pseudo-invariant scenes of playa or sand dunes.

We obtain the radiometric calibration coefficients in the hangar using a NIST-calibrated lamp and panel to direct radiances of known levels into the spectrometer. The spectral calibration, which has initially been determined in the laboratory by illuminating the spectrometer with lasers at 6–8 known wavelengths between 400 and 2500 nm, is updated from inflight data using known atmospheric spectral features. The radiometric calibration coefficients are then updated by collecting data over flat, homogenous areas where *in situ* reflectance spectra have recently been captured^41,42^. Radiometrically and spectrally calibrated data are then geolocated and orthorectified to create the Level 1B calibrated radiance product.

### Level 2A Reflectances

The primary Level 2A data product is the spectrally-resolved surface reflectance, which is related to the fraction of light reflected from the surface^45^. The AVIRIS sensors do not measure the surface reflectance value directly, rather they observe the radiance incident at the instrument aperture. Estimating the surface reflectance requires atmospheric correction: an inversion accounting for interference by gases and aerosols. These effects are insufficiently constrained by climatology so it is necessary to estimate them directly from the data.

Figure 6 summarizes the Level 2A surface reflectance product workflow. This pipeline ingests the calibrated radiances, geographic lookup table, and geolocation data produced by the L1B workflow. The surface reflectance product processing pipeline then infers the surface and atmospheric state from all channels of calibrated radiance at the sensor data for each frame. This procedure uses spectrally resolved solar radiance and atmospheric calculations for each measurement channel to infer the channel by channel reflectance and key atmospheric state parameters like the column water vapor content and aerosol optical depth. This inverse problem is mathematically under-determined as there are more unknowns than measurements, and, therefore, requires additional constraints to solve.

The AVIRIS L2A data processing workflow employs a joint probabilistic estimation of atmospheric state and surface reflectance based on Bayesian model inversion optimal estimation. This approach is a physics-based model inversion that incorporates soft prior constraints on the reflectance spectrum in specific wavelength intervals needed to make the model invertible^46^. The prior provides only weak constraints on physically plausible spectra over specific spectral intervals. The surface reflectance prior distribution reduces the effective number of degrees of freedom where most channels are uncorrelated, except for several atmospheric windows in the near-infrared region with a block-diagonal covariance that enables the separation of surface reflectance and atmospheric signals^12^. The radiative transfer model is based on sRTMnet^47^, a machine learning-based emulator of the MODTRAN radiative transfer model^48^. The optimal estimation inversion process optimizes the posterior probability of the state vector for a given measurement (frame). This approach has been validated in multiple airborne campaigns [e.g.^46^] and the EMIT mission on the international space station^12^.

### Atmospheric Correction (ISOFIT)

The open-source atmospheric correction codebase ISOFIT uses Bayesian Maximum A Posteriori (MAP) inference to simultaneously solve for the most likely surface reflectance and atmospheric state given a particular measurement. ISOFIT rigorously accounts for and propagates uncertainty, and offers the flexibility to incorporate diverse radiative transfer modeling assumptions^49^. ISOFIT optimizes a few atmospheric state parameters, including water vapor (WV), aerosol optical depth (AOD), and pressure elevation. The retrieved surface reflectance quantity represents the hemispherical-directional reflectance factor (HDRF)^50^. ISOFIT includes a superpixel segmentation and interpolation that retains fine-scale atmosphere solutions, and an analytical optimal estimation solution that preserves best-in-class spectra and uncertainties and accelerates the inversion by a factor of 20x.

## Data Records

The ABoVE AVIRIS hyperspectral imagery data records are curated by the Oak Ridge National Laboratory Distributed Active Archive Center. The 2017–2022 AVIRIS-NG data records may be freely accessed via 10.3334/ORNLDAAC/2362^51^. This dataset supersedes the previously published ABoVE AVIRIS-NG Level 2 surface reflectance files for 2017-2019^52^. AVIRIS-3 L1B calibrated radiances may be freely accessed via 10.3334/ORNLDAAC/2356^53^.

AVIRIS-NG data records for both Level 1 radiance and Level 2 surface reflectance are provided. The imagery data are provided in ENVI format along with a RGB composite image for each flight line and shapefiles showing imagery boundaries. The Complete ABoVE AVIRIS dataset includes 2,521 data files. There are 1,673 files in ENVI binary image format (compressed in *.tar.gz format), 843 RGB composite image images in JPEG or PNG format, and 5 shapefiles (compressed in *.zip archives) providing the boundaries for the imagery captured for each flight line. Level 1 (L1) radiance and Level 2 (L2) reflectance measurements are provided in separate gzip compressed TAR files, and the data are provided in ENVI format.

Files for each AVIRIS-NG flight line use a specific base filename prefix: *angYYYYMMDD*t*hhmmss*, which encodes the UTC date and time of the flight. This prefix is used for the TAR filenames and the filenames for the ENVI files contained therein. The filename prefix codes are given by

- *YYYY*: Year of flight line
- *MM*: Month of flight line
- *DD*: Day of flight line
- *hh*: UTC hour at start of flight line
- *mm*: UTC minute at start of flight line
- *ss*: UTC second at start of flight line

### File naming conventions

- L1 radiance and ancillary files: *angYYYYMMDD*t*hhmmss*.tar.gz
- L2 reflectance: *angYYYYMMDD*t*hhmmss*_rfl.tar.gz, where “rfl” denotes reflectance
- RGB composite image: *angYYYYMMDD*t*hhmmss*_RGB.jpeg or *angYYYYMMDD*t*hhmmss*_rdn_*VVVV*_img.png, where *VVVV* is the version number.
- Image boundaries: above_avirisng_boundaries_*YYYY*.zip, where *YYYY* indicates year of sampling: 2017, 2018, 2019, 2021, or 2022. These Zip archives hold shapefiles with polygons outlining image footprints.

Example file names for a single flight:

- ang20170624t181530_RGB.jpeg
- ang20170624t181530.tar.gz
- ang20170624t182330_rfl.tar.gz

### ENVI file user notes

- The binary ENVI image files have no extension and are accompanied by a header file (*.hdr) of the same name in text format. The header files should remain in the same directory as the image files for the data to be displayed properly.
- Header files provide spatial and spectral metadata related to the image file such as the number of lines, samples, channels, data ignore value, and map projection.
- No Data are coded as -9999

**L1 Radiance Files** (e.g. *ang20170817t185348.tar.gz*)

Each TAR contains six pairs of ENVI binary image files (no extension) and accompanying header files (*.hdr). In the filenames, “rdn” indicates radiance, and *VVVV* is the versioning number (e.g., “v2p9”).

1. Calibrated radiance data: angYYYYMMDDtHHNNSS_rdn_VVVV_img (&.hdr)
  Level 1 calibrated radiance from AVIRIS-NG
  Units: microwatts per centimeter squared per nanometer per steradian (µW cm^−2^ nm^−1^ sr^−1^)
  425 bands
  5-nm intervals in the visible to shortwave infrared spectral range from 380 to 2510 nm
2. Geometric lookup table (GLT): *angYYYYMMDDtHHNNSS_rdn_VVVV_glt* (&*.hdr*)
  Provides information about which original pixel occupies which output pixel in the final product
  Positive values indicate real data; negative values indicate nearest-neighbor filled data
  2 bands:
    1. Sample number
    2. Original line number
3. Input geometry file: angYYYYMMDDtHHNNSS_rdn_VVVV_igm (&.hdr)
  Contains UTM ground locations in meters for each pixel in the corresponding unorthocorrected radiance image
  See *map info* line in header file for UTM zone.
  3 bands:
    1. UTM easting (m)
    2. UTM northing (m)
    3. Estimated ground elevation at each pixel center (m)
4. Pixel location file: angYYYYMMDDtHHNNSS_rdn_VVVV_loc (&.hdr)
  Latitude/longitude pixel locations in WGS84 for each pixel in the corresponding unorthocorrected radiance image
  3 bands:
    1. WGS84 longitude (decimal degrees)
    2. WGS84 latitude (decimal degrees)
    3. Estimated ground elevation at each pixel center (m)
5. Observation parameters (unorthocorrected): *angYYYYMMDDtHHNNSS_rdn_VVVV_obs* (&*.hdr*)
  Observation parameters in the raw spatial format; matches corresponding unorthocorrected radiance image.
  11 bands:
    1. path length (sensor-to-ground in meters)
    2. to-sensor-azimuth (0 to 360 degrees clockwise from N)
    3. to-sensor-zenith (0 to 90 degrees from zenith)
    4. to-sun-azimuth (0 to 360 degrees clockwise from N)
    5. to-sun-zenith (0 to 90 degrees from zenith)
    6. solar phase (degrees between to-sensor and to-sun vectors in principal plane)
    7. slope (local surface slope as derived from DEM in degrees)
      slope values are reversed in pre-2021 data
    8. aspect (local surface aspect 0 to 360 degrees clockwise from N)
      aspect values are offset by 90 degrees in all years
      for 2022, aspect values also have additional N*360 deg offset
    9. cosine i (apparent local illumination factor based on DEM slope and aspect and to-sun vector, -1 to 1)
    10. UTC time (decimal hours for mid-line pixels)
    11. Earth-sun distance (AU)
6. Orthocorrected observation parameters: *angYYYYMMDDtHHNNSS_rdn_VVV_obs_ort* (&*.hdr*)
  Observation parameter file that has been rendered using the GLT and matches the orthocorrected imagery.
  11 bands (same as unorthocorrected; see above)
  **L2 Reflectance Files** (e.g. *ang20170817t185348_****rfl****.tar.gz*)
  Each TAR contains one ENVI binary file (no extension) and its accompanying header file (*.hdr): *angYYYYMMDDtHHNNSS_rfl* (&*.hdr*)
  Orthocorrected and atmospherically corrected surface reflectance (Gao *et al*., 1993; Thompson *et al*., 2015)
  425 bands in 5-nm intervals in the visible to shortwave infrared spectral range from 380 to 2510 nm

The 2023 AVIRIS-3 L1B data records^53^ may be freely accessed via 10.3334/ORNLDAAC/2356.

## Technical Validation

Critical evaluation of the AVIRIS data is an inherent part of the data processing and quality control^41^. Procedures optimized for the radiometric and spectral calibration of AVIRIS-NG sensor are unique. They have evolved over years of consistent effort to hone techniques and improve overall certainty, guided by quantitative feedback in regards to data product accuracy. Long-term analysis of the AVIRIS-NG calibrated radiances shows that since 2017 the flat field RMS standard deviation was 2.6 × 10^−4^, an order of magnitude improvement over pre-2017 values. Similar results are observed in the radiometric calibration coefficients where changes in the standard deviation of the calibration coefficients across calibration instances is stable to within a factor of 10^−5^. Wavelength calibration accuracy, based on fits to the 760 nm oxygen A-band and the 820 nm water absorption band, results in residual errors within the limits of AVIRIS-NG radiometric accuracy.

AVIRIS-3 performance significantly improves upon that of AVIRIS-NG, with initial calibration^42^ consistent with the instrument design parameters given in Table 2^41^. The decision to leverage inflight data for radiometric and spectral calibration has been validated: 1) Use of inflight calibration eliminates any errors associated with differences between the laboratory and in-flight calibration environments, 2) Inflight illumination levels, especially in the 380–500 nm range, can be significantly higher than those possible with most laboratory sources, leading to higher SNR and thus higher-quality calibrations for this important wavelength region, 3) Use of the same atmospheric model for calibration and data processing ensures that any errors in the model are divided out in the calibration processing, 4) Using inflight data eliminates the need for costly laboratory and onboard illumination sources and removes any sources of long-term drift, and 5) Calibration with inflight data reduces the overall cost and complexity of calibrating the instrument^42^.

Critical evaluation of AVIRIS-NG and AVIRIS-3 continues with new field campaigns [e.g.^54^] and the ongoing validation of EMIT data^11,12^. These efforts will ensure that the ABoVE AVIRIS data provide consistent year-to-year accuracy for time series investigations and may be readily intercompared with AVIRIS data records from other locations.

## Usage Notes

### File searches via base filename prefix

The ABoVE AVIRIS surveys were acquired during North American daylight savings time. In Alaska, Alaska daylight savings time, AKDT, is UTC-8, while in the Yukon and Northwest Territories, Mountain daylight savings time, MDT, is UTC-6. We note that many sorties, especially in Alaska, continued across the UTC date change threshold (0000 UTC Day N is 1600 AKDT and 1800 MDT for Day N-1). Users should not be surprised to see two specific base filename prefixes for files acquired during a single sortie. For example, ang20220731t235222 and ang20220801t000345 are valid files from the same sortie while ang20220801t201437 would represent an acquisition from a different sortie near the start of the next day’s flight. Note that both the month and day changed in the given example. Users should check that they have accessed all desired flight lines for a given day when downloading data records via prefix searches.

### Algorithms for additional L2 reflectance corrections and higher-level data product production

Many research algorithms have been developed for downstream processing to (1) Correct L2 reflectances for additional characteristics not treated by the ISOFIT processing [e.g.^44,55,56^] and (2) Retrieve higher level data products from corrected L2 reflectances. Users may find the open-source EMIT science and applications algorithms (emit-sds, https://github.com/emit-sds), EnMAP Box (https://www.enmap.org/data_tools/enmapbox/) and the python tool box from the Oak Ridge National Laboratory (https://github.com/ornldaac/above-airborne-avirisng-python) helpful. The many examples of processing imaging spectroscopy to quantify plant biodiversity collected in^57^ demonstrate the breadth of algorithms available for targeted investigations based on higher level data products derived from imaging spectroscopy data. These represent just some of the software packages that may be employed to explore the ABoVE AVIRIS data records. We note that this is not a comprehensive list, and many algorithms and processing workflows have been developed for AVIRIS and AVIRIS-class instruments.

## Data Availability

The atmospheric correction code used in this dataset is available at https://github.com/isofit/isofit/. Tutorials and documentation for using the software are available at https://github.com/isofit/isofit-tutorials/. Data used for the atmospheric correction code is available at https://github.com/isofit/isofit-data/.
